# SOD1 Protein Content in Human Central Nervous System and Peripheral Tissues

**DOI:** 10.1111/jnc.70136

**Published:** 2025-06-23

**Authors:** Laura Leykam, P. Andreas Jonsson, Karin M. E. Forsberg, Peter M. Andersen, Thomas Brännström, Stefan L. Marklund, Per Zetterström

**Affiliations:** ^1^ Department of Medical Biosciences, Clinical Chemistry Umeå University Umeå Sweden; ^2^ Department of Clinical Sciences, Neurosciences Umeå University Umeå Sweden; ^3^ Department of Public Health and Clinical Medicine Umeå University Umeå Sweden; ^4^ Department of Medical Biosciences, Pathology Umeå University Umeå Sweden

**Keywords:** ALS, amyotrophic lateral sclerosis, SOD1, SOD1 protein content

## Abstract

Gene silencing therapy is an effective treatment for amyotrophic lateral sclerosis (ALS) patients carrying mutations in the superoxide dismutase‐1 (*SOD1*) gene aiming to reduce noxious forms of SOD1 in the central nervous system (CNS). The normal steady‐state level of SOD1 protein in human CNS is therefore of interest but is contested. In this work we have analyzed SOD1 protein content, total protein content, and SOD1 enzymatic activity in six areas of the CNS as well as in four peripheral tissues from sporadic and familial ALS patients and non‐ALS controls. Our results show that SOD1 in the human CNS constitutes around 100 μg/g wet weight corresponding to about 0.16% of the total protein in the studied areas. Of the peripheral tissues analyzed, kidney and erythrocytes contain roughly equal amounts, liver higher, and skeletal muscle lower levels of SOD1 compared to the CNS. This data shows SOD1 protein levels around 10 times lower compared to previously published figures. However, SOD1 can still be considered an abundant protein considering that > 12 000 proteins are expressed in human cells. There was no difference in SOD1 protein content between sporadic or familial ALS patients and control individuals. The level and activity of SOD1 are not deviating in the areas of the CNS that are most vulnerable to ALS. Instead, insufficient control of SOD1 structure and aggregation could be important factors behind the vulnerability of motor areas to SOD1 proteotoxicity.

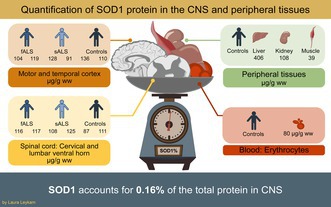

## Graphical abstract

Disease‐modifying treatments targeting the core pathological process for SOD1‐linked amyotrophic lateral sclerosis (ALS) are becoming available. Much evidence suggests that misfolding and aggregation of the SOD1 protein cause proteotoxicity, and inhibition of SOD1 expression shows a good effect in many ALS patients. We set out to analyze the SOD1 concentration and enzymatic activity in areas of the CNS and peripheral tissues in ALS patients and controls, important information for efficacy evaluation but inadequately investigated. We conclude that SOD1 makes up ~0.16% of the total protein content in the CNS. This is 10 times less than what is stated in the literature. Illustration: Laura Leykam.

**Abbreviations**
ALS: amyotrophic lateral sclerosis
ASO: antisense oligonucleotide
CNS: central nervous system
CSF: cerebrospinal fluid
CV%: coefficient of variation
fALS: familial ALS
g ww: gram wet weight
MCHC: mean corpuscular hemoglobin concentration
PBS: phosphate‐buffered saline
RRID: Research Resource Identifier (see scicrunch.org)
sALS: sporadic ALS
SD: standard deviation
SOD1: superoxide dismutase‐1
TBST: Tris‐buffered saline

## 1 Introduction

Neurodegenerative diseases have been considered as uncurable conditions with only symptomatic treatments available for many diseases. However, for some of these conditions, disease‐modifying treatments targeting the core pathological processes are becoming available, and amyotrophic lateral sclerosis (ALS) is one of those. A cause of ALS is mutations in the *superoxide dismutase‐1* (*SOD1*) gene encoding a ubiquitously expressed antioxidant enzyme. When different populations are investigated, 1%–6% of all cases, familial and sporadic disease combined, carry pathogenic mutations in *SOD1* (Benatar et al. 2025). A novel toxic gain‐of‐function is the consensus mode of action accepted for mutant SOD1 toxicity. Much evidence suggests that misfolding and aggregation that spread in a prion‐like manner trigger this toxicity (Ayers et al. 2014, 2016, 2021; Grad et al. 2011, 2014; Münch et al. 2011). Our lab has found that two structurally different strains of SOD1 aggregates can arise in five lines of transgenic mice expressing human SOD1s (Bergh et al. 2015). Both strains of SOD1 aggregates as well as SOD1 aggregates isolated from an ALS patient carrying the G127X mutation in *SOD1* show a prion‐like ability to transmit an ALS‐like disease to transgenic model mice (Bidhendi et al. 2016; Ekhtiari Bidhendi et al. 2018).

A potential treatment for ALS caused by SOD1 is to inhibit the expression of SOD1 to decrease aggregation and proteotoxicity. The antisense oligonucleotide (ASO) tofersen (Qalsody) targets the 3′‐untranslated region in the *SOD1* gene of both mutant and wildtype SOD1 and results in decreased expression with ~30% lower protein levels in the cerebrospinal fluid (CSF) of treated individuals, with reduced levels of neurofilament light chain indicating acquired neuroprotection (Miller et al. 2022). Tofersen has now been approved by the U.S. Food and Drug Administration and the European Medical Agency for the treatment of ALS patients with mutations in *SOD1*.

Since the aim of tofersen and other drugs with a similar mode of action is to lower the amount of SOD1 protein, it is important to know the steady‐state level of SOD1 in non‐treated human CNS as a reference value. Although SOD1 was identified in 1969 (McCord and Fridovich 1969) there is still a debate within the ALS community concerning the quantity of SOD1 in human tissues. SOD1 is often stated to be an abundant protein constituting up to 1%–2% of the total cellular protein in human CNS (Bakavayev et al. 2019; Banks and Andersen 2019; Bedja‐Iacona et al. 2024; Bunton‐Stasyshyn et al. 2015; Cao et al. 2008; Danzeisen et al. 2006; Duranti and Villa 2022; Eleutherio et al. 2021; Hart et al. 1998; Hasna et al. 2019; Hennig et al. 2015; Huai and Zhang 2019; Museth et al. 2009; Pardo et al. 1995; Reddi and Culotta 2013; Ricci et al. 2021; Şahin et al. 2017; Sanghai and Tranmer 2021; Seetharaman et al. 2009; Smith et al. 2006; Watson et al. 2008; Zhong et al. 2017). However, such a high concentration is not consistent with the findings in our laboratory.

In this project we set out to carefully analyze the SOD1 concentration and enzymatic activity in different areas of the CNS in sporadic and familial ALS patients and in control individuals. As a comparison we also investigated SOD1 in four peripheral tissues in the controls. We conclude that SOD1 makes up about 0.16% of the protein content in the CNS.

## 2 Material and Methods

This study was not pre‐registered since it was not a clinical trial or similar. The experimental procedures below were conducted without blinding.

### 2.1 Patient and Control Tissues

ALS patients with and without a family history of the disease were diagnosed according to the European Federation of Neurological Societies diagnostic algorithm for managing ALS (Andersen et al. 2012). For blood enzymatic and DNA analysis, with a separate written informed consent, venous blood was drawn into ethylenediaminetetraacetic acid‐containing tubes and immediately centrifuged and separated into plasma, erythrocytes, and buffy coat as described (Keskin et al. 2017). Mutation analysis of *SOD1* and 15 other ALS‐causing genes was performed as previously described (Müller et al. 2022). Patients without a family history of ALS and/or frontotemporal dementia and no known genetic mutations were considered sporadic ALS (sALS). Four patients had a family history of ALS and were considered familial ALS (fALS). Three of these four patients carried expansions in the *C9orf72* gene, and one showed no known ALS‐linked mutation. Tissue samples were collected between 1994 and 2019. The control subjects were patients with a variety of non‐motor neuron disease diagnoses such as acute myocardial infarction, pneumonia, Parkinson's disease and Huntington's disease. Tissues from 11 control subjects, 4 fALS patients, and 5 sALS patients were analyzed. A list of the included patients and controls containing sex, age at death, time to autopsy, sample storage time and cause of death is listed in Table S1. All individuals who were included had provided written informed consent, or consent was provided by the next of kin. There were no exclusion criteria, and samples from all available patients and controls were included. Tissue specimens from ALS patients and controls were immediately frozen and stored at −80°C as described (Forsberg et al. 2011, 2010). The study was performed adhering to the tenants of the 1964 Declaration of Helsinki with later amendments (WMA, 2024) and approved by the Swedish Ethical Review Authority (EPN2014‐17‐31M with later amendments).

### 2.2 Homogenization of Tissues

Cervical and lumbar spinal cord ventral horns, brain gray matter from the precentral gyrus, and superior temporal gyrus, cervical dorsal horn, corticospinal tract as well as the peripheral tissues liver, kidney, gastrocnemius muscle, and erythrocytes were analyzed. The dissected tissues (around 70–300 mg) were homogenized in 25 volumes of ice‐cold phosphate‐buffered saline (PBS, 10 mM K phosphate, pH 7.0, in 140 mM NaCl) containing an antiproteolytic cocktail (Complete with EDTA, Roche Diagnostics, Basel, Switzerland, cat. no. 1836145) with an Ultraturrax apparatus (IKA, Staufen, Germany) for 30 s, followed by pulsed sonication (1 s on/0.5 s off) during 1 min at 10% amplitude with samples on ice using a Branson Digital Sonifier SFX 250 with a 3 mm wide probe (Branson Sonifiers, Danbury, Connecticut, USA). The homogenates were stored at −80°C until analysis. Before analysis, the tissue homogenates were thawed at room temperature and sonicated again as described above, before dilutions were made for immunoblotting and total protein quantification.

### 2.3 Immunoblotting

Homogenates were made from CNS and peripheral tissue samples collected at autopsy as described above. As SOD1 standard, a human hemolysate was used, with the SOD1 content calibrated against pure human SOD1, the concentration of which was determined by quantitative amino acid analysis (Marklund et al. 1997). The tissue homogenates were diluted with PBS to match the SOD1 content of the SOD1 standard. The samples and the SOD1 standards were further diluted 1 + 1 with 2× sample buffer for SDS‐PAGE containing β‐mercaptoethanol and 10 μL of each sample were loaded on 26 well Criterion TGX Stain‐Free Any‐KD precast gels (Bio‐Rad Laboratories, Hercules, California, USA, cat. no. 56781259). The gels were run for 41 min at 200 V using a PowerPac Basic Power Supply (Bio‐Rad, cat. no. 1645050) before proteins were transferred to 0.2 μm nitrocellulose membranes using Trans‐Blot Turbo Midi Nitrocellulose transfer packs (Bio‐Rad, cat. no. 1704159) and a Trans‐Blot Turbo Transfer System (Bio‐Rad, RRID SCR_023156) using the 7 min turbo protocol for midi gels. An anti‐peptide antibody raised in rabbits immunized with a peptide corresponding to amino acids 24–39 in human SOD1 (Jonsson et al. 2004) was used as primary antibody at a concentration of 1 μg/mL diluted in 20 mM Tris‐buffered saline containing 0.1% Tween‐20 (TBST, TrizmaBase, Sigma Aldrich, St. Louis, Missouri, USA, cat. no. T1503) and 5% non‐fat dry dissolved milk powder and incubated over night at 5°C. Following washing with TBST, the filters were blocked for 90 min at room temperature in 5% non‐fat dry milk powder dissolved in TBST. The secondary antibody was an HRP‐conjugated polyclonal goat anti‐rabbit antibody (P0440 by DAKO, Santa Clara, California, USA, RRID AB2617138) incubated for 60 min at room temperature with subsequent washing with TBST. The chemiluminescent reagent was ECL Select (Cytiva, Marlborough, Massachusetts, USA, cat. no. RPN2235), and the ChemiDoc Touch Imager (Bio‐Rad, RRID SCR_019037) was used. Evaluation and quantification of the band intensities was done with the ImageLab software (Bio‐Rad, RRID SCR_014210). Three technical replicates were run for each sample. To investigate the precision of the method, homogenates of brain gray matter, lumbar ventral horn, and liver from one control individual included in the study as well as brain, spinal cord, and liver homogenates from a transgenic mouse expressing the G93A human SOD1 variant (Gurney et al. 1994) were prepared, aliquoted, and stored at −80°C until analysis. On five different days, a new set of these human and mouse homogenates and the SOD1 standard were thawed, blotted as described above, and the coefficient of variance (CV%) was calculated for the samples. The murine tissues used were from our in‐house breeding of G93AGur (G1H) mice (Gurney et al. 1994) backcrossed > 10 in C57BL/6 mice. The use and maintenance of the mice described in this article was approved by the Local Ethics Committee for Animal Research at Umeå University (A 20‐2023).

### 2.4 Total Protein Quantification

The total amount of protein present in the whole non‐centrifuged tissue homogenates was quantified using the Pierce BCA protein assay kit (ThermoFisher Scientific, Waltham, MA, USA, cat. no. 23225), following the standard protocol provided by the supplier. Pierce Bovine Serum Albumin Standard Ampules at 2 mg/mL in 0.9% NaCl (ThermoFisher Scientific, cat. no. 23209) stored at −80°C were used as the protein standard.

### 2.5 Superoxide Dismutase Activity

The SOD activity was measured with a direct spectrophotometric method as previously described, except that the assay was carried out at pH 9.5 (S. Marklund 1976). Potassium superoxide (KO_2_) (Sigma Aldrich, cat. no. 278904) is dissolved to produce superoxide anion radicals, and the SOD‐catalyzed first order dismutation of the radical is monitored at 250 nm in a LAMBDA 365 UV/Vis Spectrophotometer (PerkinElmer, Waltham, MA, USA, cat. no. N4100020). One unit is defined as the activity that brings about a dismutation at a rate of 0.1 s^−1^ in 3 mL buffer. As SOD1 standard, the same human hemolysate calibrated against pure human SOD1 (Marklund et al. 1997) used as standard for western blotting was also used for the activity measurements. SOD3 content was assayed by enzyme‐linked immunosorbent assay, and SOD3 activity was estimated by the specific activity of 8.8 ng/U as previously described (S. L. Marklund 1984). SOD2 activity was assayed with the direct spectrophotometric method after the addition of cyanide to inhibit SOD1 and SOD3. To calculate the SOD1 activity, the activities of SOD2 and SOD3 were subtracted from the total SOD activity in the sample. We have previously determined the SOD1 activity in tissue homogenates from ALS patients and controls (Jonsson et al. 2004, 2009). Most samples were still available, and the previous data were then used for calculations. When samples were unavailable, new homogenates were prepared. The homogenates were centrifuged at 5000 rpm for 10 min at room temperature in a 5417C benchtop centrifuge (Eppendorf, Hamburg, Germany) to clear the samples from particulate matter. The muscle samples had to be centrifuged twice at 14 000 rpm for 10 min at room temperature. For the measurement in blood samples, packed erythrocytes were lysed, lysates analyzed in duplicates, and the activity related to the hemoglobin content (Andersen et al. 1998; S. Marklund 1976). For the calculation of specific SOD1 activity in packed erythrocytes, an average mean corpuscular hemoglobin concentration (MCHC) of 340 mg/mL (Theodorsson et al. 2018) and a density of 1.11 g/mL (Norouzi et al. 2017) were used.

### 2.6 Statistics

Statistical analyses were done with the SPSS software (SPSS Inc., Chicago, Illinois, USA, RRID SCR_002865) version 29. Due to the limited sample size, no test for outliers was conducted. Instead, all samples were included in the statistical analysis, and the non‐parametric Kruskal–Wallis test with Bonferroni correction was used to search for differences between the controls and ALS patients, and between different organs in the controls. The significance level was set to 0.05. A full statistical report is shown in Table S3.

## 3 Results

The control individuals were 68.9 ± 14.0 years of age, the sporadic ALS patients 67.6 ± 11.7 years of age, and the familial ALS patients 60.0 ± 7.9 years of age (mean ± standard deviation [SD]) at the time of death. The time to autopsy was 50 ± 26 h for the controls, 38 ± 13 h for the sporadic ALS patients, and 36 ± 14 h for the familial ALS patients (mean ± SD). The control samples were stored 19.4 ± 9.3 years, the sporadic ALS samples 24.0 ± 4.5 years, and the familial ALS samples 26.5 ± 0.6 (mean ± SD) years before analysis of protein contents (for more information, see Table S1).

**FIGURE 1:**
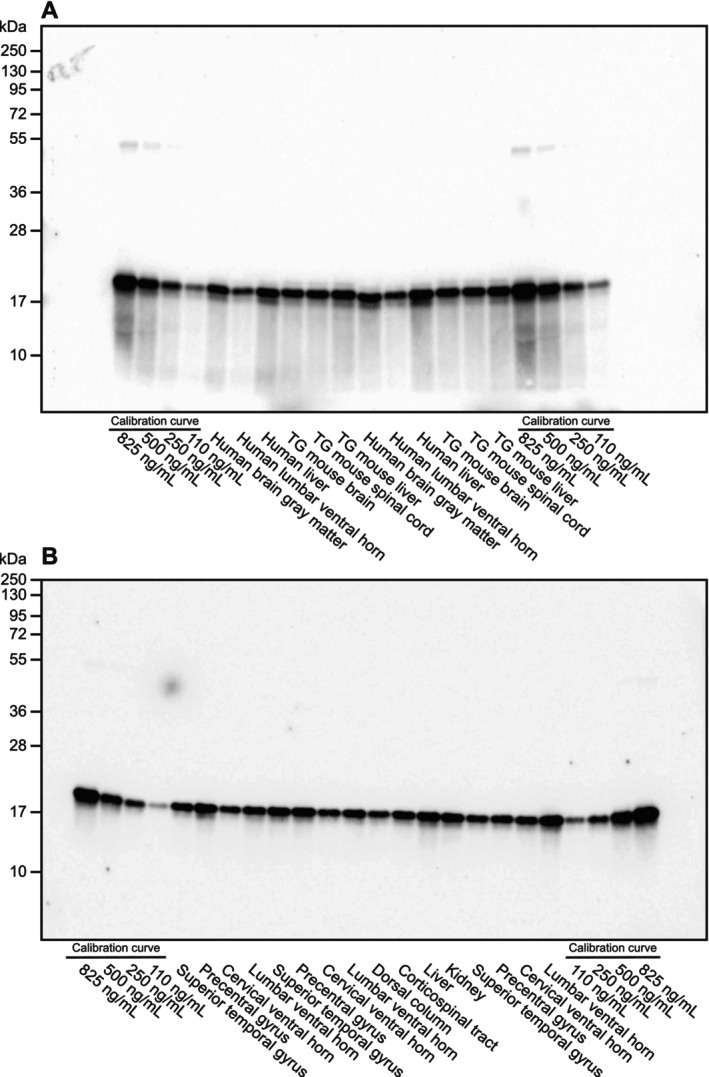
Quantification of superoxide dismutase‐1 (SOD1) protein by western immunoblot. The positions of molecular weight markers are shown (in kDa). SOD1 migrates at ~20 kDa. The faint band at ~55 kDa is a cross‐reactive band found in erythrocytes sometimes seen after long exposure times. Two SOD1 calibration curves ranging from 110 to 825 ng/mL were run on both sides of each blot. (A) A precision evaluation of the quantitative western immunoblotting method was performed during 5 days. The blots were calibrated with dilutions of a human hemolysate. Tissue homogenates from one human control subject and one transgenic mouse expressing the G93A mutation in human SOD1 were used to evaluate the method. The samples were run on five individual days, bands quantified, and the CV% calculated (see also Figure S1). One representative, uncropped gel is shown. The tissue homogenates were diluted differently to match the SOD1 content in the samples to the calibration curve. (B) A representative, uncropped western immunoblot used for quantification of SOD1 protein content in tissue samples from the included amyotrophic lateral sclerosis (ALS) patients and controls. On this particular blot, tissues from two controls, one fALS patient, and one sALS patient were analyzed. The tissue homogenates were diluted differently to match the SOD1 content in the samples to the calibration curve.

We used quantitative western immunoblotting to quantify the SOD1 content in the homogenates. To investigate the precision of the method, homogenates of brain gray matter, lumbar ventral horn, and liver from one control individual included in the study as well as brain, spinal cord, and liver homogenates from a transgenic mouse expressing the G93A human SOD1 variant (Gurney et al. 1994) were prepared, aliquoted, and stored at −80°C before they were blotted with a SOD1 calibration curve during five individual days with new aliquots of the samples and calibrators thawed each day. One representative gel is shown in Figure 1A. The coefficient of variation (CV%) was 14% in the lower end of the calibration span and 10% in the upper end (Figure S1). A combined CV% for all samples was 11% for the method. With this method, we quantified the SOD1 protein content in CNS and peripheral tissues of ALS patients and controls; a representative gel is shown in Figure 1B.

SOD1 protein content, total protein content, SOD1 activity and calculations of the fraction of total protein SOD1 accounts for as well as the specific SOD1 activity are presented in Table 1. Packed erythrocytes were collected, SOD1 activity measured at sampling, and related to the hemoglobin content. For analysis of SOD1 protein content in these samples, new aliquots were thawed, hemoglobin content re‐quantified, and SOD1 protein assessed using quantitative western immunoblot (see Section 2.3). The results are shown in Table 1. The contents of SOD2 and SOD3 used to calculate the SOD1 activity are presented in Table S2.

**TABLE 1:** Values for protein and superoxide dismutase‐1 (SOD1) activity in controls and amyotrophic lateral sclerosis (ALS) patients.

	SOD1 (μg/g ww)			Total protein (μg/g ww)			Fraction SOD1 (%)			SOD1 activity (U/g ww)^a^			Specific SOD1 activity (ng/U)		
	Controls	sALS	fALS	Controls	sALS	fALS	Controls	sALS	fALS	Controls	sALS	fALS	Controls	sALS	fALS
Cervical ventral horn	87 ± 30	106 ± 22	116 ± 4	68 400 ± 15 000	59 200 ± 7300	62 900 ± 11 400	0.14 ± 0.06	0.18 ± 0.02	0.19 ± 0.03	12 300 ± 3200	11 800 ± 2100	12 400 ± 1400	7.7 ± 4.0	9.1 ± 1.3	9.5 ± 1.2
Lumbar ventral horn	111 ± 18	125 ± 15	118 ± 5	64 700 ± 7400	63 000 ± 6200	63 800 ± 3100	0.17 ± 0.03	0.20 ± 0.03	0.18 ± 0.01	14 600 ± 1400	14 500 ± 1400	16 500 ± 2700	7.6 ± 0.6	8.8 ± 1.8	7.2 ± 0.9
Precentral gyrus	137 ± 29	129 ± 27	105 ± 30	73 400 ± 9000	81 200 ± 14 600	77 100 ± 5000	0.19 ± 0.03	0.16 ± 0.04	0.14 ± 0.04	14 400 ± 1600	14 800 ± 800	16 100 ± 7600	9.5 ± 1.7	8.6 ± 1.6	7.1 ± 1.7
Superior temporal gyrus	110 ± 30	91 ± 2	120 ± 27	78 300 ± 28 500	66 300 ± 11 000	69 900 ± 8100	0.16 ± 0.07	0.14 ± 0.02	0.17 ± 0.03	14 500 ± 3100	15 400 ± 1400	16 200 ± 2900	7.5 ± 0.9	6.0 ± 0.6	7.3 ± 0.5
Cervical dorsal horn	92 ± 40	97		58 900 ± 11 600	67 700		0.15 ± 0.04	0.14		*13 900 ± 3900*	13 500		*7.5 ± 0.7*	*7.2*	
Corticospinal tract	84 ± 39	105		55 000 ± 7200	68 700		0.15 ± 0.06	0.15		*12 600 ± 1100*	15 500		*7.9 ± 2.9*	*6.8*	
Liver	406 ± 39			143 500 ± 2800			0.28 ± 0.03			61 000 ± 13 200			6.8 ± 0.9		
Kidney	108 ± 23			77 300 ± 12 100			0.14 ± 0.01			13 700 ± 5000			8.4 ± 2.2		
Skeletal muscle	39 ± 11			64 900 ± 20 700			0.06 ± 0.02			5500 ± 900			7.0 ± 1.4		

	SOD1 (μg/g ww)^c^	SOD1 activity (U/mg Hb)	Specific SOD1 activity (ng/U)						
	Controls	Controls	Controls						
Packed erythrocytes^b^	80 ± 5	48 ± 6	5.4 ± 0.6						

*Note:* Values are means ± standard deviation. For controls *n* = 5 except superior temporal gyrus, cervical dorsal horn, corticospinal tract, and skeletal muscle where *n* = 4. Italics, *n* = 3. For sALS *n* = 5 except cervical ventral horn where *n* = 4 and cervical dorsal horn and corticospinal tract where *n* = 1. For fALS *n* = 4 except cervical ventral horn where *n* = 3.

^a^ Data mainly adopted from Jonsson et al. (2004, 2009). If no material was available for SOD1 protein quantification by western immunoblot, new homogenates were prepared and analyzed.

^b^ For packed erythrocytes, controls *n* = 10.

^c^ Calculated from average mean corpuscular hemoglobin concentration (MCHC) = 340 mg/mL (Theodorsson et al. 2018) and a density of 1.11 (Norouzi et al. 2017).

**FIGURE 2:**
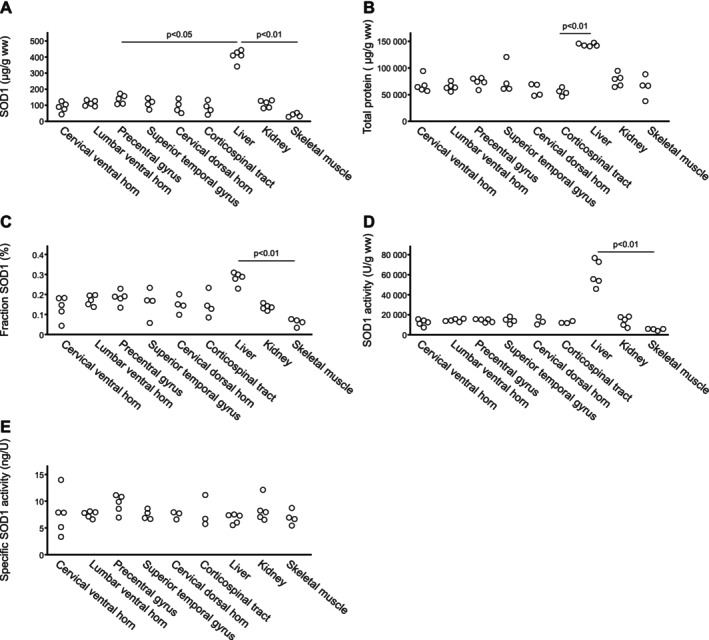
Scatter plot of protein content in the controls. Each dot represents a single control subject, and all included subjects are shown. Differences between different organs were assed with the Kruskal–Wallis test with Bonferroni correction and all statistically significant (*p* < 0.05) differences identified are shown in the figure. (A) Superoxide dismutase‐1 (SOD1) protein content in different central nervous system (CNS) and peripheral tissues. The liver contained significantly higher SOD1 protein levels compared to the precentral gyrus (*p* < 0.05) and muscle (*p* < 0.01). (B) Total protein content in different CNS and peripheral tissues. The total protein content in the liver was significantly higher compared to the corticospinal tract (*p* < 0.01). (C) SOD1 protein fraction of total protein content in different CNS and peripheral tissues. The SOD1 fraction was higher in the liver compared to muscle (*p* < 0.01). (D) SOD1 activity in different CNS and peripheral tissues. The SOD1 activity was higher in liver compared to muscle (*p* < 0.01). (E) Specific SOD1 activity (the amount of SOD1 protein producing one unit U of SOD1 enzymatic activity) in different CNS and peripheral tissues.

There were no statistically significant differences (*p* > 0.05) between the controls and ALS patients in the CNS tissues except for a lower specific SOD1 activity in the superior temporal gyrus in the sALS group compared to the controls and fALS patients (both *p* = 0.039). Differences between the CNS areas and peripheral tissues were calculated in the controls since no data was available for peripheral tissues in the ALS patients. In the controls, the liver contains more SOD1 and has a higher SOD1 activity compared to the other organs, but due to the limited sample size, only a few differences were statistically significant (Figure 2). For comparison, all the ALS patients are plotted together with the controls in Figure S2.

## 4 Discussion

Oxidative stress is a source of damage to cellular components such as proteins, lipids, and DNA and may cause severe injury in many cells such as mutagenesis and decreased enzymatic function. Different cellular systems are designed to protect the cell from harm caused by various free radical species. Among these, the superoxide dismutases are evolutionarily conserved to be the primary scavenging system for the superoxide anion radical. There are three isoforms expressed in mammals, with SOD1 being the primary cytosolic isoenzyme, SOD2 located in the mitochondrial matrix, and SOD3 found in the extracellular space.

The defense against free radicals could be the reason for the relatively high abundance of SOD1 in most cells. Following this, SOD1 is often stated to be a very abundant protein forming ~1%–2% of all cellular protein (Bakavayev et al. 2019; Banks and Andersen 2019; Bunton‐Stasyshyn et al. 2015; Cao et al. 2008; Danzeisen et al. 2006; Duranti and Villa 2022; Eleutherio et al. 2021; Hart et al. 1998; Hasna et al. 2019; Hennig et al. 2015; Huai and Zhang 2019; Museth et al. 2009; Pardo et al. 1995; Reddi and Culotta 2013; Ricci et al. 2021; Şahin et al. 2017; Sanghai and Tranmer 2021; Seetharaman et al. 2009; Smith et al. 2006; Watson et al. 2008; Zhong et al. 2017). Hennig et al. state that SOD1 constitutes 1%–2% of the cellular protein in multiple cell types (Hennig et al. 2015). However, no data or reference is provided for this figure, but this paper is still cited as a source for SOD1 concentration (Bedja‐Iacona et al. 2024; Eleutherio et al. 2021). The most frequently cited source (Bakavayev et al. 2019; Banks and Andersen 2019; Bunton‐Stasyshyn et al. 2015; Cao et al. 2008; Danzeisen et al. 2006; Duranti and Villa 2022; Hart et al. 1998; Hasna et al. 2019; Huai and Zhang 2019; Museth et al. 2009; Reddi and Culotta 2013; Ricci et al. 2021; Şahin et al. 2017; Sanghai and Tranmer 2021; Seetharaman et al. 2009; Smith et al. 2006; Watson et al. 2008; Zhong et al. 2017) is likely a publication by Pardo et al. who found SOD1 to constitute up to 2% of the detergent‐insoluble protein in the CNS (Pardo et al. 1995). Here we used quantitative immunoblot calibrated against pure SOD1, the concentration of which was determined by quantitative amino acid analysis (Marklund et al. 1997) to quantify SOD1 with high precision. In control individuals, we find the SOD1 concentration to be ~80–130 μg/g wet weight in CNS tissues corresponding to on average 0.16% of the total tissue protein (Table 1). This is considerably lower compared to the figure reported by Pardo et al. There could be several explanations for the difference observed: (i) Pardo et al. analyzed SOD1 as a fraction of the detergent‐soluble protein in 1% SDS; here we quantified all tissue protein; (ii) different antibodies are used for SOD1 detection; (iii) only one sample was quantified by Pardo et al.; and (iv) the calibration of the internal standard is different. We also compared SOD1 in CNS tissues from sporadic and familial ALS patients, all lacking mutations in SOD1. The SOD1 levels in the CNS did not differ significantly from controls except in the superior temporal lobe that normally is spared in ALS. Three of the familial cases carried expansions in *C9orf72* implying that there are no differences in SOD1 content in this group of patients compared to controls and sporadic patients. Also, SOD1 has previously been quantified in rat tissues with SOD1 levels and concentration similar to our findings in humans. Rat brain contained 93 μg/g tissue which corresponds to ~0.1% of the cellular protein content (Asayama and Burr 1985).

We used a hemolysate from a healthy donor as an internal SOD1 standard both for the quantitative western immunoblots and for the activity measurements. The SOD1 content and activity of the internal standard were carefully calibrated against pure SOD1 as described above. A potential confounding factor could be that SOD1 in erythrocytes carries different posttranslational modifications compared to SOD1 in CNS and peripheral tissues, and this could influence the results. Several posttranslational modifications have been identified in SOD1 (Bedja‐Iacona et al. 2024) but when isolated from erythrocytes, SOD1 was only found to be partially modified by acetylation, phosphorylation, and glutathionylation (Marklund et al. 1997; Wilcox et al. 2009). The antibody used for the quantitative western immunoblots is directed towards amino acids 24–39 in SOD1, a region that may carry posttranslational modifications (Bedja‐Iacona et al. 2024). However, since the antibody used is a polyclonal serum, it will likely detect modified SOD1 as well. Still, different patterns of posttranslational modifications in different CNS regions and peripheral tissues compared to erythrocytes could have influenced the result.

The current study is the largest effort to quantify the SOD1 protein in human postmortem tissues. We conclude that SOD1 constitutes 0.14%–0.19% of the cellular protein in the CNS. Since the human genome consists of ~20 000 genes with around 12 000 quantifiable proteins in different tissues (Jiang et al. 2020) SOD1 is still expressed at a rate ~2000 times higher than if all 12 000 cellular proteins were to be equally expressed. Therefore, SOD1 can still be termed an abundant protein.

There is a larger variation of SOD1 protein amounts in peripheral tissues compared to within the CNS. The kidney contains a similar amount of SOD1 as the CNS, about 100 μg/g wet weight, whereas the liver contains higher amounts and skeletal muscles lower amounts compared to the CNS (406 and 39 μg/g wet weight corresponding to 0.28% and 0.06% of the tissue protein, respectively). CSF contains even less, with only ~0.03% of the total protein content consisting of SOD1 (Leykam et al. 2024), which is less than the fraction in CNS tissues. However, since 80% of the protein in CSF derives from plasma (Thompson and Keir 1990), SOD1 accounts for about 0.15% of the CNS‐derived proteins in CSF, very similar to the fraction found in the CNS (Table 1). Differences in SOD1 content and activity likely reflect the reactive oxygen species burden in different tissues. The specific SOD1 activity is instead an indicator of the folding status of SOD1 and was found to be similar in all tissues investigated, with values ~8 ng/U (Table 1). SOD1 in erythrocytes shows a higher specific activity (5.4 ng/U), similar to the previously published specific activity of 4.8 ng/U in fully Cu‐ and Zn‐charged SOD1 (Andersen et al. 1998). Hence, there is a fraction of inactive SOD1 present in all organs analyzed. However, we could not detect any significant differences between the tissues, possibly due to our limited sample size.

The SOD1 content is uniform over the CNS areas examined with no large differences in the motor areas of the CNS that are most affected by ALS. SOD1 knockdown by tofersen and other SOD1 protein‐reducing agents that are non‐allele specific will affect all areas of the CNS in a similar manner, including those which are less impaired during ALS disease progression, given a similar distribution throughout the CNS after intrathecal injection of the drug. The risk of extra‐CNS side effects is limited due to the intrathecal delivery.

We here found no differences in SOD1 expression between various parts of the CNS that could explain the specific vulnerability of motor areas to mutant SOD1 toxicity. Nor were there any differences between controls and ALS patients without SOD1 mutations. In mice, the SOD1 expression is similar in brain and spinal cord, but the aggregation of the protein and neuron loss is much more evident in the spinal cord in transgenic mice expressing mutant human SOD1s (Bruijn et al. 1997; Gurney et al. 1994; Jonsson et al. 2004, 2006; Turner and Talbot 2008; Wang et al. 2002). We previously homogenized fresh (within 30 min after dissection) non‐frozen murine tissues from the CNS and from the periphery in buffer containing a thiol‐blocking agent to prevent artificial oxidation of the C57–C146 disulfide bond. We found a marked enrichment of disordered mutant human SOD1 monomers in spinal cord compared to the brain and even less in the peripheral tissues (Zetterstrom et al. 2013, 2007). Such analyses cannot be made with human autopsy tissues, but we have found low levels of several important autophagy factors in the lamina IX of the ventral horn compared to other areas of the CNS (Tokuda et al. 2016). Thus, insufficient control of SOD1 structure and aggregation could be important factors behind the vulnerability of motor areas to SOD1‐linked neurotoxicity. Enhanced clearance of misfolded and aggregated proteins via activation of autophagy could be a promising complementing therapeutic intervention to combat ALS.

## Author Contributions

**Laura Leykam:** investigation, formal analysis, visualization, writing – review and editing. **P. Andreas Jonsson:** investigation, writing – review and editing. **Karin M. E. Forsberg:** resources, writing – review and editing. **Peter M. Andersen:** writing – review and editing, resources, formal analysis, funding acquisition. **Thomas Brännström:** resources, writing – review and editing, funding acquisition. **Stefan L. Marklund:** conceptualization, methodology, supervision, formal analysis, writing – review and editing. **Per Zetterström:** conceptualization, project administration, visualization, writing – review and editing, writing – original draft, funding acquisition, formal analysis.

## Ethics Statement

The study was approved by the Swedish Ethical Review Authority (FEK 1994 dnr 94‐135 with later amendments). The approval includes permission to collect, store, and analyze samples for human molecular and genetic ALS research and to publish the results in a scientific journal in such a way that no individuals results can be identified. Informed consent was obtained from participants or their next of kin. The use and maintenance of the mice described in this article were approved by the Local Ethics Committee for Animal Research at Umeå University.

## Conflicts of Interest

L.L., P.A.J., T.B., S.L.M., and P.Z.: The authors declare no conflicts of interest. K.M.E.F.: Clinical trial site investigator for Amylyx, Biogen, Ionis Pharmaceuticals, PTC Pharmaceuticals, Sanofi, and ITB‐Med. P.M.A.: Paid consultancies and serve/have served on advisory boards for Biogen, Roche, Arrowhead, Avrion, Regeneron, uniQure, Voyager, and Orphazyme A/S; clinical trial site investigator for AB Science, AL‐S Pharma, and Lilly, Amylyx, Alexion Pharmaceuticals, Biogen Idec, IBT‐Med, IONIS Pharmaceuticals, Orion Pharma, PTC Pharmaceuticals, and Sanofi. Since 1993, Director of the ALS‐genetic laboratory at Umeå University Hospital that performs not‐for‐profit research genetic testing and free genetic testing, including for SOD1. Since 2021, member of the ClinGen ALS Gene variant Curation Expert panel. External advisor to the European Medicine Agency.

## Peer Review

The peer review history for this article is available at https://www.webofscience.com/api/gateway/wos/peer‐review/10.1111/jnc.70136.

## Supporting information


Data S1.

## Data Availability

The data that support the findings of this study are available from the corresponding author upon reasonable request, if of scientific interest and legally and ethically possible.
