# Supplementary material for: SOD1 Protein Content in Human Central Nervous System and Peripheral Tissues
Source: J Neurochem. 2025 Jun 23;169(6):e70136. doi: 10.1111/jnc.70136 (PMC12184320; doi:10.1111/jnc.70136)
Supplement: Supplementary file 1 — Data S1. [file JNC-169-0-s001.pdf]

Supplementary material for

SOD1 protein content in human central nervous system and peripheral tissues

Laura Leykam[1], P. Andreas Jonsson[1], Karin M.E. Forsberg[2], Peter M. Andersen[2], Thomas Brännström[1], Stefan L. Marklund[1], Per Zetterström[1*]

[1] Department of Medical Biosciences, Clinical Chemistry, Umeå University, S-901 85, Umeå, Sweden.

[2] Department of Clinical Sciences, Neurosciences, Umeå University, S-901 85 Umeå, Sweden.

* To whom correspondence should be addressed:

Per Zetterström

Department of Medical Biosciences, Clinical Chemistry

Umeå University

SE-901 85 Umeå, SWEDEN

Tel: +46 90 785 2950

Per.Zetterstrom@umu.se

Figure S1

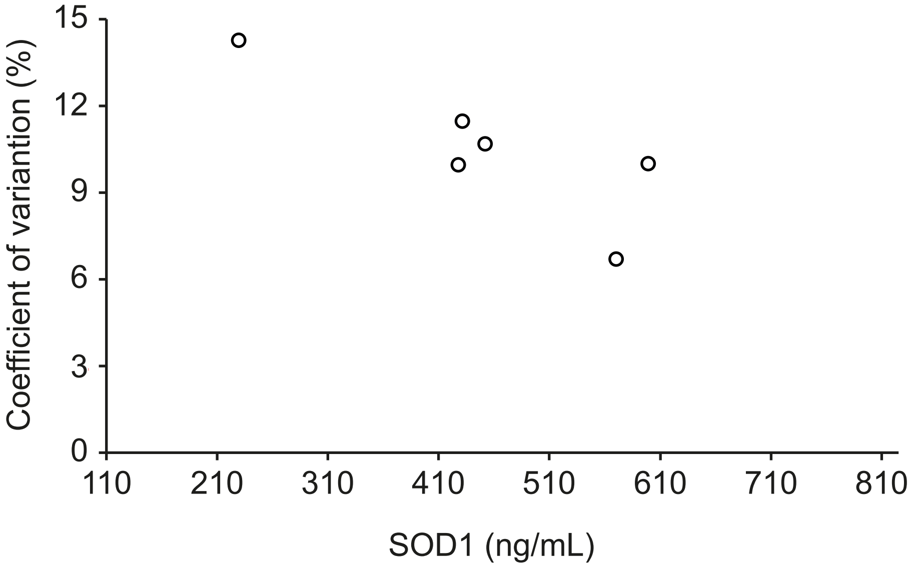

Figure S1: Precision of quantitative western immunoblot. To investigate the precision of the method, homogenates of brain grey matter, lumbar ventral horn, and liver from n=1 control individual not included in the study as well as brain, spinal cord, and liver homogenates from n=1 transgenic mouse expressing the G93A human SOD1 variant (Gurney et al., 1994) were prepared, aliquoted, and stored at −80 °C until analysis. On five different days, a new set of these human and mouse homogenates and the SOD1 standard was thawed, blotted as described, and the coefficient of variance (CV%) were calculated for the samples. The calculated CV% of all six samples are plotted against the SOD1 content of the sample. The X-axis covers the full range of the SOD1 calibration curve used (110-825 ng/mL). The imprecision is higher at the lower end of the calibration curve.

Figure S2

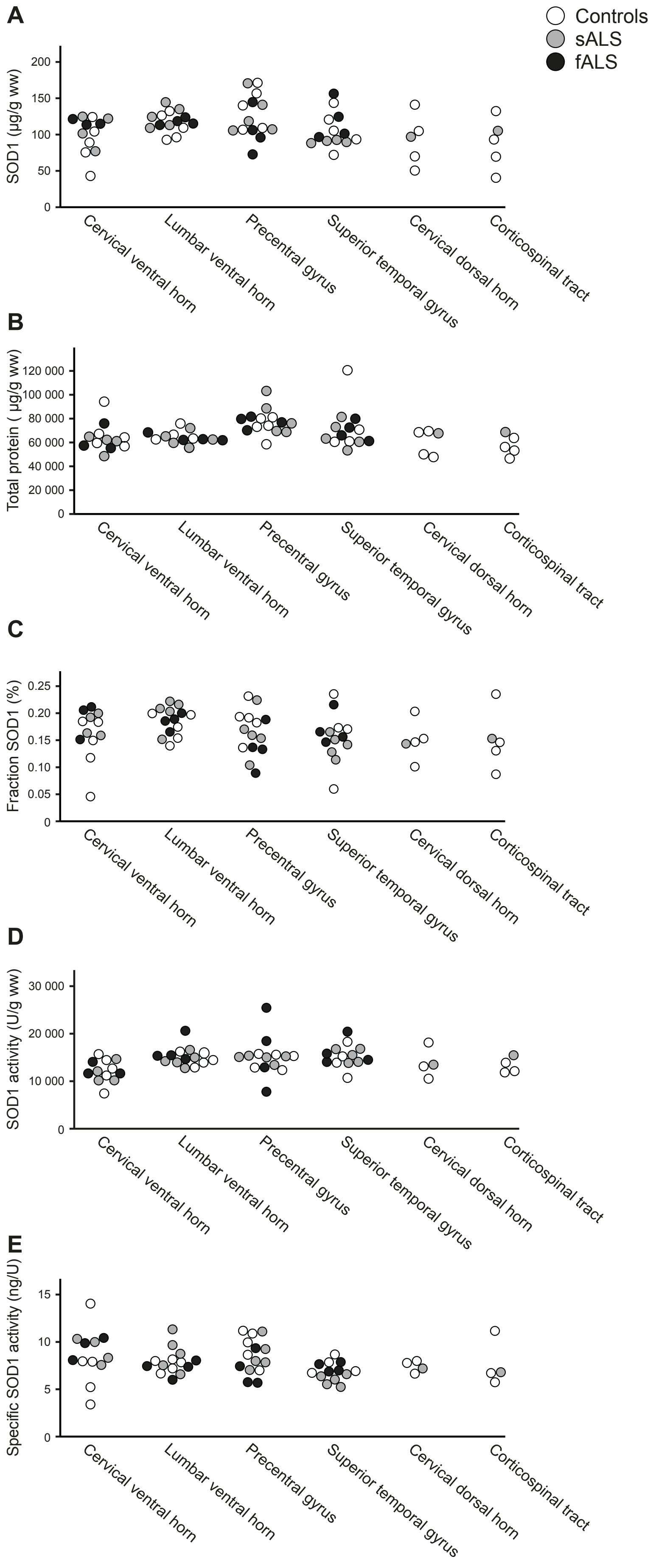
A
Controls
sALS
fALS
SOD1 (µg/g ww)
200
150
100
50
0
Cervical ventral horn
Lumbar ventral horn
Precentral gyrus
Superior temporal gyrus
Cervical dorsal horn
Corticospinal tract
B
Total protein (µg/g ww)
120 000
100 000
80 000
60 000
40 000
20 000
0
Cervical ventral horn
Lumbar ventral horn
Precentral gyrus
Superior temporal gyrus
Cervical dorsal horn
Corticospinal tract
C
Fraction SOD1 (%)
0.25
0.20
0.15
0.10
0.05
0
Cervical ventral horn
Lumbar ventral horn
Precentral gyrus
Superior temporal gyrus
Cervical dorsal horn
Corticospinal tract
D
SOD1 activity (U/g ww)
30 000
20 000
10 000
0
Cervical ventral horn
Lumbar ventral horn
Precentral gyrus
Superior temporal gyrus
Cervical dorsal horn
Corticospinal tract
E
Specific SOD1 activity (ng/U)
15
10
5
0
Cervical ventral horn
Lumbar ventral horn
Precentral gyrus
Superior temporal gyrus
Cervical dorsal horn
Corticospinal tract

Figure S2: Scatter plot of protein content in the controls and ALS patients. Each dot represents a single subject, and all included subjects are shown. Means and standard deviation for the control, sALS, and fALS groups are found in Table 1. A) SOD1 protein content in different CNS and peripheral tissues. B) Total protein content in different CNS and peripheral tissues. C) SOD1 protein fraction of total protein content in different CNS and peripheral tissues. D) SOD1 activity in different CNS and peripheral tissues. E) Specific SOD1 activity (the amount of SOD1 protein producing one unit U of SOD1 enzymatic activity) in different CNS and peripheral tissues.

Table S1. Information about included individuals.

Individual	Disease	Sex	Age at death (years)	Time to autopsy (h)	Storage time (years)	Cause of death	Available samples
1	control	Male	54	48	25	Acute myocardial infraction	DH; CST
2	control	Male	66	24	25	N.a.	CVH; LVH; PG; STG; DH; CST
3	control	Male	43	24	27	Huntington's disease	CVH; LVH; PG; STG; DH; CST
5	control	Female	80	48	27	Acute myocardial infraction	CVH; LVH; PG
5	control	Female	64	48	27	Pulmonary infraction	CVH; LVH; PG; STG; DH; CST
6	control	Male	58	48	27	Dissecting aortic aneurysm	CVH; LVH; PG; STG
7	control	Male	64	24	16	Bronchopneumonia	Liver; Kidney; SM
8	control	Female	88	30	21	Acute myocardial infraction	Liver; Kidney
9	control	Male	79	72	3	Parkinson's disease	Liver; Kidney; SM
10	control	Male	82	96	8	Parkinson's disease	Liver; Kidney; SM
11	control	Male	80	96	8	Parkinson's disease	Liver; Kidney; SM
12	fALS	Male	64	48	27	ALS	LVH; PG; STG
13	fALS	Female	55	24	26	ALS	CVH; LVH; PG; STG
14	fALS	Male	52	24	27	ALS	CVH; LVH; PG; STG
15	fALS	Male	69	48	26	ALS	CVH; LVH; PG; STG
16	sALS	Female	65	24	26	ALS	CVH; LVH; PG; STG
17	sALS	Male	50	24	26	ALS	LVH; PG; STG; DH; CST
18	sALS	Male	69	44	26	ALS	CVH; LVH; PG; STG
19	sALS	Female	82	48	26	ALS	CVH; LVH; PG; STG
20	sALS	Female	72	52	16	ALS	CVH; LVH; PG; STG

Abbreviations: CST, corticospinal tract; CVH, cervical ventral horn; DH, cervical dorsal horn; fALS, familial ALS; LVH, lumbar ventral horn; N.a., not available; PG, precentral gyrus; sALS, sporadic ALS; SM, skeletal muscle; STG, superior temporal gyrus.

Table S2. SOD2 and SOD3 activity in controls and ALS patients.

	SOD2 (U/g ww)			SOD3 (U/g ww)		
	Controls	sALS	fALS	Controls	sALS	fALS
Cervical ventral horn	471 ± 121	526 ± 101	712 ± 250	130 ± 102	113 ± 61	147 ± 80
Lumbar ventral horn	800 ± 220	811 ± 272	1 074 ± 690	101 ± 59	125 ± 82	74 ± 24
Precentral gyrus	942 ± 376	918 ± 117	1154 ± 410	102 ± 33	70 ± 14	92 ± 19
Superior temporal gyrus	853 ± 117	833 ± 128	919 ± 259	97 ± 24	88 ± 26	100 ± 29
Cervical dorsal horn	522 ± 63	491		132 ± 47	96	
Corticospinal tract	435 ± 159	1 008		157 ± 62	286	
Liver	8 438 ± 4 546			90 ± 34		
Kidney	3 128 ± 911			1 323 ± 1 214		
Skeletal muscle	507 ± 527			818 ± 75		

Values are means ± standard deviation.
For control n=5 except superior temporal gyrus, cervical dorsal horn, cortical spinal tract, liver, kidney, and skeletal muscle where n = 4.
For sALS n=5 except cervical ventral horn where n=4. For dorsal horn and corticospinal tract, n = 1.
For fALS n=4 except cervical ventral horn where n=3.
Data mainly adopted from Jonsson *et al* (19). If no material was available for SOD1 protein quantification by western blot, new homogenates were prepared and analyzed.

Table S3: Full statistical report

Kruskal-Wallis test for differences between patients and controls in Cervical ventral horn

Hypothesis Test Summary, Independent-Samples Kruskal-Wallis Test

Null Hypothesis	Sig.[a,b]	Decision
1. The distribution of SOD1Protein is the same across categories of Disease.	.379	Retain the null hypothesis.
2. The distribution of TotalProtein is the same across categories of Disease.	.671	Retain the null hypothesis.
3. The distribution of PercentSOD1 is the same across categories of Disease.	.151	Retain the null hypothesis.
4. The distribution of SOD1Activityt is the same across categories of Disease.	.871	Retain the null hypothesis.
5. The distribution of SpecificSOD1Actgivity is the same across categories of Disease.	.357	Retain the null hypothesis.

a. The significance level is ,050.

b. Asymptotic significance is displayed.

SOD1 protein in Cervical Ventral Horn

Independent-Samples Kruskal-Wallis Test Summary

Total N	12
Test Statistic	1.938[a]
Degree Of Freedom	2
Asymptotic Sig.(2-sided test)	.379

a. The test statistic is adjusted for ties.

Pairwise Comparisons of SOD1 protein in Cervical Ventral Horn

Sample 1-Sample 2	Test Statistic	Std. Error	Std. Test Statistic	Sig.	Adj. Sig.[a]
Control-sALS	-2.700	2.419	-1.116	.264	.793
Control-fALS	-3.200	2.633	-1.215	.224	.673
sALS-fALS	-.500	2.754	-.182	.856	1.000

Each row tests the null hypothesis that the Sample 1 and Sample 2 distributions are the same.
Asymptotic significances (2-sided tests) are displayed. The significance level is ,050.
a. Significance values have been adjusted by the Bonferroni correction for multiple tests.

Total protein in Cervical Ventral Horn

Independent-Samples Kruskal-Wallis Test Summary

Total N	12
Test Statistic	.799[a]
Degree Of Freedom	2
Asymptotic Sig.(2-sided test)	.671

a. The test statistic is adjusted for ties.

Pairwise Comparisons of total protein in Cervical Ventral Horn

Sample 1-Sample 2	Test Statistic	Std. Error	Std. Test Statistic	Sig.	Adj. Sig.[a]
fALS-sALS	.083	2.754	.030	.976	1.000
fALS-Control	1.933	2.633	.734	.463	1.000
sALS-Control	1.850	2.419	.765	.444	1.000

Each row tests the null hypothesis that the Sample 1 and Sample 2 distributions are the same.
Asymptotic significances (2-sided tests) are displayed. The significance level is ,050.

a. Significance values have been adjusted by the Bonferroni correction for multiple tests.

Fraction SOD1 protein in Cervical Ventral Horn

Independent-Samples Kruskal-Wallis Test Summary

Total N	12
Test Statistic	3.785[a]
Degree Of Freedom	2
Asymptotic Sig.(2-sided test)	.151

a. The test statistic is adjusted for ties.

Pairwise Comparisons of fraction SOD1 protein in Cervical Ventral Horn

Sample 1-Sample 2	Test Statistic	Std. Error	Std. Test Statistic	Sig.	Adj. Sig.[a]
Control-sALS	-3.300	2.419	-1.364	.172	.517
Control-fALS	-4.800	2.633	-1.823	.068	.205
sALS-fALS	-1.500	2.754	-.545	.586	1.000

Each row tests the null hypothesis that the Sample 1 and Sample 2 distributions are the same.
Asymptotic significances (2-sided tests) are displayed. The significance level is ,050.
a. Significance values have been adjusted by the Bonferroni correction for multiple tests.

SOD1 activity in Cervical Ventral Horn

Independent-Samples Kruskal-Wallis Test Summary

Total N	12
Test Statistic	.276[a]
Degree Of Freedom	2
Asymptotic Sig.(2-sided test)	.871

a. The test statistic is adjusted for ties.

Pairwise Comparisons of SOD1 activity in Cervical Ventral Horn

Sample 1-Sample 2	Test Statistic	Std. Error	Std. Test Statistic	Sig.	Adj. Sig.[a]
sALS-fALS	-.917	2.754	-.333	.739	1.000
sALS-Control	1.250	2.419	.517	.605	1.000
fALS-Control	.333	2.633	.127	.899	1.000

Each row tests the null hypothesis that the Sample 1 and Sample 2 distributions are the same.
Asymptotic significances (2-sided tests) are displayed. The significance level is ,050.
a. Significance values have been adjusted by the Bonferroni correction for multiple tests.

Specific SOD1 activity in Cervical Ventral Horn

Independent-Samples Kruskal-Wallis Test Summary

Total N	12
Test Statistic	2.060[a]
Degree Of Freedom	2
Asymptotic Sig.(2-sided test)	.357

a. The test statistic is adjusted for ties.

Pairwise Comparisons of specific SOD1 activity in Cervical Ventral Horn

Sample 1-Sample 2	Test Statistic	Std. Error	Std. Test Statistic	Sig.	Adj. Sig.[a]
Control-sALS	-2.450	2.419	-1.013	.311	.933
Control-fALS	-3.533	2.633	-1.342	.180	.539
sALS-fALS	-1.083	2.754	-.393	.694	1.000

Each row tests the null hypothesis that the Sample 1 and Sample 2 distributions are the same.

Asymptotic significances (2-sided tests) are displayed. The significance level is ,050.
a. Significance values have been adjusted by the Bonferroni correction for multiple tests.

Kruskal-Wallis test for differences between patients and controls in Lumbar Ventral Horn

Hypothesis Test Summary, Independent-Samples Kruskal-Wallis Test

Null Hypothesis		
1. The distribution of SOD1Protein is the same across categories of Disease.	Sig.[a,b]	Decision
2.The distribution of TotalProtein is the same across categories of Disease.	.526	Retain the null hypothesis.
3. The distribution of PercentSOD1 is the same across categories of Disease.	.825	Retain the null hypothesis.
4. The distribution of SOD1Activityt is the same across categories of Disease.	.132	Retain the null hypothesis.
5. The distribution of SpecificSOD1Actgivity is the same across categories of Disease.	.359	Retain the null hypothesis.
a. The significance level is ,050.	.325	Retain the null hypothesis.

b. Asymptotic significance is displayed.

SOD1 protein in Lumbar ventral Horn

Independent-Samples Kruskal-Wallis Test Summary

Total N	14
Test Statistic	1.286[a]
Degree Of Freedom	2
Asymptotic Sig.(2-sided test)	.526

a. The test statistic is adjusted for ties.

Pairwise Comparisons of SOD1 protein in Lumbar ventral Horn

Sample 1-Sample 2	Test Statistic	Std. Error	Std. Test Statistic	Sig.	Adj. Sig.[a]
Control-fALS	-1.500	2.806	-.535	.593	1.000
Control-sALS	-3.000	2.646	-1.134	.257	.771
fALS-sALS	1.500	2.806	.535	.593	1.000

Each row tests the null hypothesis that the Sample 1 and Sample 2 distributions are the same.
Asymptotic significances (2-sided tests) are displayed. The significance level is ,050.
a. Significance values have been adjusted by the Bonferroni correction for multiple tests.

Total protein in Lumbar ventral Horn

Independent-Samples Kruskal-Wallis Test Summary

Total N	14
Test Statistic	.386[a]
Degree Of Freedom	2
Asymptotic Sig.(2-sided test)	.825

a. The test statistic is adjusted for ties.

Pairwise Comparisons of total protein in Lumbar ventral Horn

Sample 1-Sample 2	Test Statistic	Std. Error	Std. Test Statistic	Sig.	Adj. Sig.[a]
sALS-fALS	-.450	2.806	-.160	.873	1.000
sALS-Control	1.600	2.646	.605	.545	1.000
fALS-Control	1.150	2.806	.410	.682	1.000

Each row tests the null hypothesis that the Sample 1 and Sample 2 distributions are the same.
Asymptotic significances (2-sided tests) are displayed. The significance level is ,050.
a. Significance values have been adjusted by the Bonferroni correction for multiple tests.

Fraction SOD1 protein in Lumbar ventral Horn

Independent-Samples Kruskal-Wallis Test Summary

Total N	14
Test Statistic	4.043[a]
Degree Of Freedom	2
Asymptotic Sig.(2-sided test)	.132

a. The test statistic is adjusted for ties.

Pairwise Comparisons of fraction SOD1 protein in Lumbar ventral Horn

Sample 1-Sample 2	Test Statistic	Std. Error	Std. Test Statistic	Sig.	Adj. Sig.[a]
Control-fALS	-1.550	2.806	-.552	.581	1.000
Control-sALS	-5.200	2.646	-1.965	.049	.148
fALS-sALS	3.650	2.806	1.301	.193	.580

Each row tests the null hypothesis that the Sample 1 and Sample 2 distributions are the same.
Asymptotic significances (2-sided tests) are displayed. The significance level is ,050.
a. Significance values have been adjusted by the Bonferroni correction for multiple tests.

SOD1 activity in Lumbar ventral Horn

Independent-Samples Kruskal-Wallis Test Summary

Total N	14
Test Statistic	2.051[a]
Degree Of Freedom	2
Asymptotic Sig.(2-sided test)	.359

a. The test statistic is adjusted for ties.

Pairwise Comparisons of SOD1 activity in Lumbar ventral Horn

Sample 1-Sample 2	Test Statistic	Std. Error	Std. Test Statistic	Sig.	Adj. Sig.[a]
sALS-Control	.600	2.646	.227	.821	1.000
sALS-fALS	-3.800	2.806	-1.354	.176	.527
Control-fALS	-3.200	2.806	-1.140	.254	.762

Each row tests the null hypothesis that the Sample 1 and Sample 2 distributions are the same.
Asymptotic significances (2-sided tests) are displayed. The significance level is ,050.
a. Significance values have been adjusted by the Bonferroni correction for multiple tests.

Specific SOD1 activity in Lumbar ventral Horn

Independent-Samples Kruskal-Wallis Test Summary

Total N	14
Test Statistic	2.246[a]
Degree Of Freedom	2
Asymptotic Sig.(2-sided test)	.325

a. The test statistic is adjusted for ties.

Pairwise Comparisons of specific SOD1 activity in Lumbar ventral Horn

Sample 1-Sample 2	Test Statistic	Std. Error	Std. Test Statistic	Sig.	Adj. Sig.[a]
fALS-Control	1.500	2.806	.535	.593	1.000
fALS-sALS	4.100	2.806	1.461	.144	.432
Control-sALS	-2.600	2.646	-.983	.326	.977

Each row tests the null hypothesis that the Sample 1 and Sample 2 distributions are the same.
Asymptotic significances (2-sided tests) are displayed. The significance level is ,050.
a. Significance values have been adjusted by the Bonferroni correction for multiple tests.

Kruskal-Wallis test for differences between patients and controls in Precentral gyrus

Hypothesis Test Summary, Independent-Samples Kruskal-Wallis Test

Null Hypothesis		
1. The distribution of SOD1Protein is the same across categories of Disease.	Sig.[a,b]	Decision
2. The distribution of TotalProtein is the same across categories of Disease.	.206	Retain the null hypothesis.
3. The distribution of PercentSOD1 is the same across categories of Disease.	.825	Retain the null hypothesis.
4. The distribution of SOD1Activityt is the same across categories of Disease.	.170	Retain the null hypothesis.
5. The distribution of SpecificSOD1Actgivity is the same across categories of Disease.	.958	Retain the null hypothesis.
a. The significance level is ,050.	.188	Retain the null hypothesis.

b. Asymptotic significance is displayed.

SOD1 protein in Precentral gyrus

Independent-Samples Kruskal-Wallis Test Summary

Total N	14
Test Statistic	3.160[a]
Degree Of Freedom	2
Asymptotic Sig.(2-sided test)	.206

a. The test statistic is adjusted for ties.

Pairwise Comparisons of SOD1 protein in Precentral gyrus

Sample 1-Sample 2	Test Statistic	Std. Error	Std. Test Statistic	Sig.	Adj. Sig.[a]
fALS-sALS	3.500	2.806	1.247	.212	.637
fALS-Control	4.900	2.806	1.746	.081	.242
sALS-Control	1.400	2.646	.529	.597	1.000

Each row tests the null hypothesis that the Sample 1 and Sample 2 distributions are the same.
Asymptotic significances (2-sided tests) are displayed. The significance level is ,050.
a. Significance values have been adjusted by the Bonferroni correction for multiple tests.

Total protein in Precentral gyrus

Independent-Samples Kruskal-Wallis Test Summary

Total N	14
Test Statistic	.386[a]
Degree Of Freedom	2
Asymptotic Sig.(2-sided test)	.825

a. The test statistic is adjusted for ties.

Pairwise Comparisons of total protein in Precentral gyrus

Sample 1-Sample 2	Test Statistic	Std. Error	Std. Test Statistic	Sig.	Adj. Sig.[a]
Control-sALS	-1.200	2.646	-.454	.650	1.000
Control-fALS	-1.650	2.806	-.588	.557	1.000
sALS-fALS	-.450	2.806	-.160	.873	1.000

Each row tests the null hypothesis that the Sample 1 and Sample 2 distributions are the same.
Asymptotic significances (2-sided tests) are displayed. The significance level is ,050.
a. Significance values have been adjusted by the Bonferroni correction for multiple tests.

Fraction SOD1 protein in Precentral gyrus

Independent-Samples Kruskal-Wallis Test Summary

Total N	14
Test Statistic	3.540[a]
Degree Of Freedom	2
Asymptotic Sig.(2-sided test)	.170

a. The test statistic is adjusted for ties.

Pairwise Comparisons of fraction SOD1 protein in Precentral gyrus

Sample 1-Sample 2	Test Statistic	Std. Error	Std. Test Statistic	Sig.	Adj. Sig.a
fALS-sALS	2.450	2.806	.873	.383	1.000
fALS-Control	5.250	2.806	1.871	.061	.184
sALS-Control	2.800	2.646	1.058	.290	.870

Each row tests the null hypothesis that the Sample 1 and Sample 2 distributions are the same.
Asymptotic significances (2-sided tests) are displayed. The significance level is ,050.
a. Significance values have been adjusted by the Bonferroni correction for multiple tests.

SOD1 activity in Precentral gyrus

Independent-Samples Kruskal-Wallis Test Summary

Total N	14
Test Statistic	.086[a]
Degree Of Freedom	2
Asymptotic Sig.(2-sided test)	.958

a. The test statistic is adjusted for ties.

Pairwise Comparisons of SOD1 activity in Precentral gyrus

Sample 1-Sample 2	Test Statistic	Std. Error	Std. Test Statistic	Sig.	Adj. Sig.a
sALS-Control	.200	2.646	.076	.940	1.000
sALS-fALS	-.800	2.806	-.285	.776	1.000
Control-fALS	-.600	2.806	-.214	.831	1.000

Each row tests the null hypothesis that the Sample 1 and Sample 2 distributions are the same.
Asymptotic significances (2-sided tests) are displayed. The significance level is ,050.
a. Significance values have been adjusted by the Bonferroni correction for multiple tests.

Specific SOD1 activity in Precentral gyrus

Independent-Samples Kruskal-Wallis Test Summary

Total N	14
Test Statistic	3.343[a]
Degree Of Freedom	2
Asymptotic Sig.(2-sided test)	.188

a. The test statistic is adjusted for ties.

Pairwise Comparisons of specific SOD1 activity in Precentral gyrus

Sample 1-Sample 2	Test Statistic	Std. Error	Std. Test Statistic	Sig.	Adj. Sig.a
fALS-sALS	3.300	2.806	1.176	.240	.719
fALS-Control	5.100	2.806	1.817	.069	.207
sALS-Control	1.800	2.646	.680	.496	1.000

Each row tests the null hypothesis that the Sample 1 and Sample 2 distributions are the same.
Asymptotic significances (2-sided tests) are displayed. The significance level is ,050.
a. Significance values have been adjusted by the Bonferroni correction for multiple tests.

Kruskal-Wallis test for differences between patients and controls in Superial temporal gyrus

Hypothesis Test Summary, Independent-Samples Kruskal-Wallis Test

Null Hypothesis		
1.The distribution of SOD1Protein is the same across categories of Disease.	Sig.[a,b]	Decision
2. The distribution of TotalProtein is the same across categories of Disease.	.073	Retain the null hypothesis.
3. The distribution of PercentSOD1 is the same across categories of Disease.	.789	Retain the null hypothesis.
4. The distribution of SOD1Activityt is the same across categories of Disease.	.210	Retain the null hypothesis.
5. The distribution of SpecificSOD1Actgivity is the same across categories of Disease.	.709	Retain the null hypothesis.
a. The significance level is ,050.	.014	Reject the null hypothesis.

b. Asymptotic significance is displayed.

SOD1 protein in Superior temporal gyrus

Independent-Samples Kruskal-Wallis Test Summary

Total N	13
Test Statistic	5.225[a]
Degree Of Freedom	2
Asymptotic Sig.(2-sided test)	.073

a. The test statistic is adjusted for ties.

Pairwise Comparisons of SOD1 protein in Superior temporal gyrus

Sample 1-Sample 2	Test Statistic	Std. Error	Std. Test Statistic	Sig.	Adj. Sig.[a]
sALS-Control	4.000	2.612	1.531	.126	.377
sALS-fALS	-5.750	2.612	-2.201	.028	.083
Control-fALS	-1.750	2.754	-.635	.525	1.000

Each row tests the null hypothesis that the Sample 1 and Sample 2 distributions are the same.
Asymptotic significances (2-sided tests) are displayed. The significance level is ,050.
a. Significance values have been adjusted by the Bonferroni correction for multiple tests.

Total protein in Superior temporal gyrus

Independent-Samples Kruskal-Wallis Test Summary

Total N	13
Test Statistic	.475[a]
Degree Of Freedom	2
Asymptotic Sig.(2-sided test)	.789

a. The test statistic is adjusted for ties.

Pairwise Comparisons of total protein in Superior temporal gyrus

Sample 1-Sample 2	Test Statistic	Std. Error	Std. Test Statistic	Sig.	Adj. Sig.[a]
sALS-Control	.800	2.612	.306	.759	1.000
sALS-fALS	-1.800	2.612	-.689	.491	1.000
Control-fALS	-1.000	2.754	-.363	.717	1.000

Each row tests the null hypothesis that the Sample 1 and Sample 2 distributions are the same.
Asymptotic significances (2-sided tests) are displayed. The significance level is ,050.
a. Significance values have been adjusted by the Bonferroni correction for multiple tests.

Fraction SOD1 protein in Superior temporal gyrus

Independent-Samples Kruskal-Wallis Test Summary

Total N	13
Test Statistic	3.119[a]
Degree Of Freedom	2
Asymptotic Sig.(2-sided test)	.210

a. The test statistic is adjusted for ties.

Pairwise Comparisons of fraction SOD1 protein in Superior temporal gyrus

Sample 1-Sample 2	Test Statistic	Std. Error	Std. Test Statistic	Sig.	Adj. Sig.[a]
sALS-fALS	-3.650	2.612	-1.397	.162	.487
sALS-Control	4.150	2.612	1.589	.112	.336
fALS-Control	.500	2.754	.182	.856	1.000

Each row tests the null hypothesis that the Sample 1 and Sample 2 distributions are the same.
Asymptotic significances (2-sided tests) are displayed. The significance level is ,050.
a. Significance values have been adjusted by the Bonferroni correction for multiple tests.

SOD1 activity in Superior temporal gyrus

Independent-Samples Kruskal-Wallis Test Summary

Total N	13
Test Statistic	.689[a]
Degree Of Freedom	2
Asymptotic Sig.(2-sided test)	.709

a. The test statistic is adjusted for ties.

Pairwise Comparisons of SOD1 activity in Superior temporal gyrus

Sample 1-Sample 2	Test Statistic	Std. Error	Std. Test Statistic	Sig.	Adj. Sig.[a]
Control-sALS	-1.450	2.612	-.555	.579	1.000
Control-fALS	-2.250	2.754	-.817	.414	1.000
sALS-fALS	-.800	2.612	-.306	.759	1.000

Each row tests the null hypothesis that the Sample 1 and Sample 2 distributions are the same.
Asymptotic significances (2-sided tests) are displayed. The significance level is ,050.
a. Significance values have been adjusted by the Bonferroni correction for multiple tests.

Specific SOD1 activity in Superior temporal gyrus

Independent-Samples Kruskal-Wallis Test Summary

Total N	13
Test Statistic	8.571[a]
Degree Of Freedom	2
Asymptotic Sig.(2-sided test)	.014

a. The test statistic is adjusted for ties.

Pairwise Comparisons of specific SOD1 activity in Superior temporal gyrus

Sample 1-Sample 2	Test Statistic	Std. Error	Std. Test Statistic	Sig.	Adj. Sig.[a]
sALS-Control	6.500	2.612	2.488	.013	.039
sALS-fALS	-6.500	2.612	-2.488	.013	.039
Control-fALS	.000	2.754	.000	1.000	1.000

Each row tests the null hypothesis that the Sample 1 and Sample 2 distributions are the same.
Asymptotic significances (2-sided tests) are displayed. The significance level is ,050.
a. Significance values have been adjusted by the Bonferroni correction for multiple tests.

Kruskal-Wallis test for differences between tissues in controls

Hypothesis Test Summary, Independent-Samples Kruskal-Wallis Test

Null Hypothesis		
1. The distribution of SOD1Protein is the same across categories of Tissue.	Sig.[a,b]	Decision
2. The distribution of TotalProtein is the same across categories of Tissue.	.001	Reject the null hypothesis.
3. The distribution of PercentSOD1 is the same across categories of Tissue.	.007	Reject the null hypothesis.
4. The distribution of SOD1Activityt is the same across categories of Tissue.	.004	Reject the null hypothesis.
5. The distribution of SpecificSOD1Activity is the same across categories of Tissue.	.004	Reject the null hypothesis.
a. The significance level is .050.	.482	Retain the null hypothesis.

b. Asymptotic significance is displayed.

SOD1 protein in controls

Independent-Samples Kruskal-Wallis Test Summary

Total N	41
Test Statistic	25.302[a]
Degree Of Freedom	8
Asymptotic Sig.(2-sided test)	.001

a. The test statistic is adjusted for ties.

Pairwise Comparisons of SOD1 protein in controls

Sample 1-Sample 2	Test Statistic	Std. Error	Std. Test Statistic	Sig.	Adj. Sig.a
Skeletal muscle-Corticospinal tract	11.000	8.471	1.299	.194	1.000
Skeletal muscle-Cervical ventral horn	11.300	8.036	1.406	.160	1.000
Skeletal muscle-Cervical dorsal horn	13.250	8.471	1.564	.118	1.000
Skeletal muscle-Kidney	17.700	8.036	2.203	.028	.994
Skeletal muscle-Superial temporal gyrus	18.750	8.471	2.214	.027	.967
Skeletal muscle-Lumbar ventral horn	18.900	8.036	2.352	.019	.672
Skeletal muscle-Precentral gyrus	25.700	8.036	3.198	.001	.050
Skeletal muscle-Liver	35.500	8.036	4.418	<.001	.000
Corticospinal tract-Cervical ventral horn	.300	8.036	.037	.970	1.000
Corticospinal tract-Cervical dorsal horn	2.250	8.471	.266	.791	1.000
Corticospinal tract-Kidney	-6.700	8.036	-.834	.404	1.000
Corticospinal tract-Superial temporal gyrus	7.750	8.471	.915	.360	1.000
Corticospinal tract-Lumbar ventral horn	7.900	8.036	.983	.326	1.000
Corticospinal tract-Precentral gyrus	14.700	8.036	1.829	.067	1.000
Corticospinal tract-Liver	-24.500	8.036	-3.049	.002	.083
Cervical ventral horn-Cervical dorsal horn	-1.950	8.036	-.243	.808	1.000
Cervical ventral horn-Kidney	-6.400	7.576	-.845	.398	1.000
Cervical ventral horn-Superial temporal gyrus	-7.450	8.036	-.927	.354	1.000
Cervical ventral horn-Lumbar ventral horn	-7.600	7.576	-1.003	.316	1.000
Cervical ventral horn-Precentral gyrus	-14.400	7.576	-1.901	.057	1.000
Cervical ventral horn-Liver	-24.200	7.576	-3.194	.001	.050
Cervical dorsal horn-Kidney	-4.450	8.036	-.554	.580	1.000
Cervical dorsal horn-Superial temporal gyrus	5.500	8.471	.649	.516	1.000
Cervical dorsal horn-Lumbar ventral horn	5.650	8.036	.703	.482	1.000
Cervical dorsal horn-Precentral gyrus	12.450	8.036	1.549	.121	1.000
Cervical dorsal horn-Liver	-22.250	8.036	-2.769	.006	.203
Kidney-Superial temporal gyrus	1.050	8.036	.131	.896	1.000

Kidney-Lumbar ventral horn	1.200	7.576	.158	.874	1.000
Kidney-Precentral gyrus	8.000	7.576	1.056	.291	1.000
Kidney-Liver	17.800	7.576	2.349	.019	.677
Superial temporal gyrus-Lumbar ventral horn	.150	8.036	.019	.985	1.000
Superial temporal gyrus-Precentral gyrus	6.950	8.036	.865	.387	1.000
Superial temporal gyrus-Liver	-16.750	8.036	-2.084	.037	1.000
Lumbar ventral horn-Precentral gyrus	-6.800	7.576	-.898	.369	1.000
Lumbar ventral horn-Liver	-16.600	7.576	-2.191	.028	1.000
Precentral gyrus-Liver	-9.800	7.576	-1.294	.196	1.000

Each row tests the null hypothesis that the Sample 1 and Sample 2 distributions are the same.
Asymptotic significances (2-sided tests) are displayed. The significance level is ,050.
a. Significance values have been adjusted by the Bonferroni correction for multiple tests.

Total protein in controls

Independent-Samples Kruskal-Wallis Test Summary

Total N	41
Test Statistic	21.217[a]
Degree Of Freedom	8
Asymptotic Sig.(2-sided test)	.007

a. The test statistic is adjusted for ties.

Pairwise Comparisons of total protein in controls

Sample 1-Sample 2	Test Statistic	Std. Error	Std. Test Statistic	Sig.	Adj. Sig.a
Corticospinal tract-Cervical dorsal horn	6.250	8.471	.738	.461	1.000
Corticospinal tract-Lumbar ventral horn	8.550	8.036	1.064	.287	1.000
Corticospinal tract-Cervical ventral horn	10.750	8.036	1.338	.181	1.000
Corticospinal tract-Skeletal muscle	-11.000	8.471	-1.299	.194	1.000
Corticospinal tract-Superial temporal gyrus	13.750	8.471	1.623	.105	1.000
Corticospinal tract-Precentral gyrus	17.350	8.036	2.159	.031	1.000
Corticospinal tract-Kidney	-19.550	8.036	-2.433	.015	.539
Corticospinal tract-Liver	-31.750	8.036	-3.951	<.001	.003
Cervical dorsal horn-Lumbar ventral horn	2.300	8.036	.286	.775	1.000
Cervical dorsal horn-Cervical ventral horn	4.500	8.036	.560	.575	1.000
Cervical dorsal horn-Skeletal muscle	-4.750	8.471	-.561	.575	1.000
Cervical dorsal horn-Superial temporal gyrus	7.500	8.471	.885	.376	1.000
Cervical dorsal horn-Precentral gyrus	11.100	8.036	1.381	.167	1.000
Cervical dorsal horn-Kidney	-13.300	8.036	-1.655	.098	1.000
Cervical dorsal horn-Liver	-25.500	8.036	-3.173	.002	.054
Lumbar ventral horn-Cervical ventral horn	2.200	7.576	.290	.772	1.000
Lumbar ventral horn-Skeletal muscle	-2.450	8.036	-.305	.760	1.000
Lumbar ventral horn-Superial temporal gyrus	-5.200	8.036	-.647	.518	1.000
Lumbar ventral horn-Precentral gyrus	-8.800	7.576	-1.162	.245	1.000
Lumbar ventral horn-Kidney	-11.000	7.576	-1.452	.147	1.000
Lumbar ventral horn-Liver	-23.200	7.576	-3.062	.002	.079
Cervical ventral horn-Skeletal muscle	-.250	8.036	-.031	.975	1.000
Cervical ventral horn-Superial temporal gyrus	-3.000	8.036	-.373	.709	1.000
Cervical ventral horn-Precentral gyrus	-6.600	7.576	-.871	.384	1.000
Cervical ventral horn-Kidney	-8.800	7.576	-1.162	.245	1.000
Cervical ventral horn-Liver	-21.000	7.576	-2.772	.006	.201
Skeletal muscle-Superial temporal gyrus	2.750	8.471	.325	.745	1.000
Skeletal muscle-Precentral gyrus	6.350	8.036	.790	.429	1.000

Skeletal muscle-Kidney	8.550	8.036	1.064	.287	1.000
Skeletal muscle-Liver	20.750	8.036	2.582	.010	.353
Superial temporal gyrus-Precentral gyrus	3.600	8.036	.448	.654	1.000
Superial temporal gyrus-Kidney	-5.800	8.036	-.722	.470	1.000
Superial temporal gyrus-Liver	-18.000	8.036	-2.240	.025	.903
Precentral gyrus-Kidney	-2.200	7.576	-.290	.772	1.000
Precentral gyrus-Liver	-14.400	7.576	-1.901	.057	1.000
Kidney-Liver	12.200	7.576	1.610	.107	1.000

Each row tests the null hypothesis that the Sample 1 and Sample 2 distributions are the same.
Asymptotic significances (2-sided tests) are displayed. The significance level is ,050.
a. Significance values have been adjusted by the Bonferroni correction for multiple tests.

Fraction SOD1 protein in controls

Independent-Samples Kruskal-Wallis Test Summary

Total N	41
Test Statistic	22.618[a]
Degree Of Freedom	8
Asymptotic Sig.(2-sided test)	.004

a. The test statistic is adjusted for ties.

Pairwise Comparisons of fraction SOD1 protein in controls

Sample 1-Sample 2	Test Statistic	Std. Error	Std. Test Statistic	Sig.	Adj. Sig.a
Skeletal muscle-Kidney	10.800	8.036	1.344	.179	1.000
Skeletal muscle-Cervical ventral horn	13.000	8.036	1.618	.106	1.000
Skeletal muscle-Corticospinal tract	13.750	8.471	1.623	.105	1.000
Skeletal muscle-Cervical dorsal horn	15.750	8.471	1.859	.063	1.000
Skeletal muscle-Superial temporal gyrus	17.750	8.471	2.095	.036	1.000
Skeletal muscle-Lumbar ventral horn	20.800	8.036	2.588	.010	.347
Skeletal muscle-Precentral gyrus	22.400	8.036	2.788	.005	.191
Skeletal muscle-Liver	34.600	8.036	4.306	<.001	.001
Kidney-Cervical ventral horn	2.200	7.576	.290	.772	1.000
Kidney-Corticospinal tract	2.950	8.036	.367	.714	1.000
Kidney-Cervical dorsal horn	4.950	8.036	.616	.538	1.000
Kidney-Superial temporal gyrus	6.950	8.036	.865	.387	1.000
Kidney-Lumbar ventral horn	10.000	7.576	1.320	.187	1.000
Kidney-Precentral gyrus	11.600	7.576	1.531	.126	1.000
Kidney-Liver	23.800	7.576	3.141	.002	.061
Cervical ventral horn-Corticospinal tract	-.750	8.036	-.093	.926	1.000
Cervical ventral horn-Cervical dorsal horn	-2.750	8.036	-.342	.732	1.000
Cervical ventral horn-Superial temporal gyrus	-4.750	8.036	-.591	.554	1.000
Cervical ventral horn-Lumbar ventral horn	-7.800	7.576	-1.030	.303	1.000
Cervical ventral horn-Precentral gyrus	-9.400	7.576	-1.241	.215	1.000
Cervical ventral horn-Liver	-21.600	7.576	-2.851	.004	.157
Corticospinal tract-Cervical dorsal horn	2.000	8.471	.236	.813	1.000
Corticospinal tract-Superial temporal gyrus	4.000	8.471	.472	.637	1.000
Corticospinal tract-Lumbar ventral horn	7.050	8.036	.877	.380	1.000
Corticospinal tract-Precentral gyrus	8.650	8.036	1.076	.282	1.000
Corticospinal tract-Liver	-20.850	8.036	-2.595	.009	.341
Cervical dorsal horn-Superial temporal gyrus	2.000	8.471	.236	.813	1.000
Cervical dorsal horn-Lumbar ventral horn	5.050	8.036	.628	.530	1.000
Cervical dorsal horn-Precentral gyrus	6.650	8.036	.828	.408	1.000

Cervical dorsal horn-Liver	-18.850	8.036	-2.346	.019	.684
Superial temporal gyrus-Lumbar ventral horn	3.050	8.036	.380	.704	1.000
Superial temporal gyrus-Precentral gyrus	4.650	8.036	.579	.563	1.000
Superial temporal gyrus-Liver	-16.850	8.036	-2.097	.036	1.000
Lumbar ventral horn-Precentral gyrus	-1.600	7.576	-.211	.833	1.000
Lumbar ventral horn-Liver	-13.800	7.576	-1.821	.069	1.000
Precentral gyrus-Liver	-12.200	7.576	-1.610	.107	1.000

Each row tests the null hypothesis that the Sample 1 and Sample 2 distributions are the same.
Asymptotic significances (2-sided tests) are displayed. The significance level is ,050.
a. Significance values have been adjusted by the Bonferroni correction for multiple tests.

SOD1 activity in controls

Independent-Samples Kruskal-Wallis Test Summary

Total N	39
Test Statistic	22.667a
Degree Of Freedom	8
Asymptotic Sig.(2-sided test)	.004

a. The test statistic is adjusted for ties.

Pairwise Comparisons of SOD1 activity in controls

Sample 1-Sample 2	Test Statistic	Std. Error	Std. Test Statistic	Sig.	Adj. Sig.a
Skeletal muscle-Corticospinal tract	11.167	8.708	1.282	.200	1.000
Skeletal muscle-Cervical ventral horn	13.300	7.649	1.739	.082	1.000
Skeletal muscle-Dorsal column	16.500	8.708	1.895	.058	1.000
Skeletal muscle-Kidney	17.700	7.649	2.314	.021	.744
Skeletal muscle-Superial temporal gyrus	18.500	8.062	2.295	.022	.783
Skeletal muscle-Precentral gyrus	18.900	7.649	2.471	.013	.485
Skeletal muscle-Lumbar ventral horn	20.700	7.649	2.706	.007	.245
Skeletal muscle-Liver	34.500	7.649	4.511	<.001	.000
Corticospinal tract-Cervical ventral horn	2.133	8.327	.256	.798	1.000
Corticospinal tract-Dorsal column	5.333	9.309	.573	.567	1.000
Corticospinal tract-Kidney	-6.533	8.327	-.785	.433	1.000
Corticospinal tract-Superial temporal gyrus	7.333	8.708	.842	.400	1.000
Corticospinal tract-Precentral gyrus	7.733	8.327	.929	.353	1.000
Corticospinal tract-Lumbar ventral horn	9.533	8.327	1.145	.252	1.000
Corticospinal tract-Liver	-23.333	8.327	-2.802	.005	.183
Cervical ventral horn-Dorsal column	-3.200	8.327	-.384	.701	1.000
Cervical ventral horn-Kidney	-4.400	7.211	-.610	.542	1.000
Cervical ventral horn-Superial temporal gyrus	-5.200	7.649	-.680	.497	1.000
Cervical ventral horn-Precentral gyrus	-5.600	7.211	-.777	.437	1.000
Cervical ventral horn-Lumbar ventral horn	-7.400	7.211	-1.026	.305	1.000
Cervical ventral horn-Liver	-21.200	7.211	-2.940	.003	.118
Dorsal column-Kidney	-1.200	8.327	-.144	.885	1.000
Dorsal column-Superial temporal gyrus	2.000	8.708	.230	.818	1.000
Dorsal column-Precentral gyrus	2.400	8.327	.288	.773	1.000
Dorsal column-Lumbar ventral horn	4.200	8.327	.504	.614	1.000
Dorsal column-Liver	-18.000	8.327	-2.162	.031	1.000
Kidney-Superial temporal gyrus	.800	7.649	.105	.917	1.000
Kidney-Precentral gyrus	1.200	7.211	.166	.868	1.000
Kidney-Lumbar ventral horn	3.000	7.211	.416	.677	1.000
Kidney-Liver	16.800	7.211	2.330	.020	.714

Superial temporal gyrus-Precentral gyrus	.400	7.649	.052	.958	1.000
Superial temporal gyrus-Lumbar ventral horn	2.200	7.649	.288	.774	1.000
Superial temporal gyrus-Liver	-16.000	7.649	-2.092	.036	1.000
Precentral gyrus-Lumbar ventral horn	1.800	7.211	.250	.803	1.000
Precentral gyrus-Liver	-15.600	7.211	-2.163	.031	1.000
Lumbar ventral horn-Liver	-13.800	7.211	-1.914	.056	1.000

Each row tests the null hypothesis that the Sample 1 and Sample 2 distributions are the same.
Asymptotic significances (2-sided tests) are displayed. The significance level is .050.
a. Significance values have been adjusted by the Bonferroni correction for multiple tests.

Specific SOD1 activity in controls

Independent-Samples Kruskal-Wallis Test Summary

Total N	39
Test Statistic	7.522a
Degree Of Freedom	8
Asymptotic Sig.(2-sided test)	.482

a. The test statistic is adjusted for ties.

Pairwise Comparisons of specific SOD1 activity in controls

Sample 1-Sample 2	Test Statistic	Std. Error	Std. Test Statistic	Sig.	Adj. Sig.a
Liver-Skeletal muscle	-1.850	7.649	-.242	.809	1.000
Liver-Corticospinal tract	3.600	8.327	.432	.665	1.000
Liver-Cervical ventral horn	4.800	7.211	.666	.506	1.000
Liver-Cervical dorsal horn	4.933	8.327	.592	.554	1.000
Liver-Superial temporal gyrus	6.350	7.649	.830	.406	1.000
Liver-Lumbar ventral horn	7.600	7.211	1.054	.292	1.000
Liver-Kidney	-10.400	7.211	-1.442	.149	1.000
Liver-Precentral gyrus	17.000	7.211	2.357	.018	.662
Skeletal muscle-Corticospinal tract	1.750	8.708	.201	.841	1.000
Skeletal muscle-Cervical ventral horn	2.950	7.649	.386	.700	1.000
Skeletal muscle-Cervical dorsal horn	3.083	8.708	.354	.723	1.000
Skeletal muscle-Superial temporal gyrus	4.500	8.062	.558	.577	1.000
Skeletal muscle-Lumbar ventral horn	5.750	7.649	.752	.452	1.000
Skeletal muscle-Kidney	8.550	7.649	1.118	.264	1.000
Skeletal muscle-Precentral gyrus	15.150	7.649	1.981	.048	1.000
Corticospinal tract-Cervical ventral horn	1.200	8.327	.144	.885	1.000
Corticospinal tract-Cervical dorsal horn	1.333	9.309	.143	.886	1.000
Corticospinal tract-Superial temporal gyrus	2.750	8.708	.316	.752	1.000
Corticospinal tract-Lumbar ventral horn	4.000	8.327	.480	.631	1.000
Corticospinal tract-Kidney	-6.800	8.327	-.817	.414	1.000
Corticospinal tract-Precentral gyrus	13.400	8.327	1.609	.108	1.000
Cervical ventral horn-Cervical dorsal horn	-.133	8.327	-.016	.987	1.000
Cervical ventral horn-Superial temporal gyrus	-1.550	7.649	-.203	.839	1.000
Cervical ventral horn-Lumbar ventral horn	-2.800	7.211	-.388	.698	1.000
Cervical ventral horn-Kidney	-5.600	7.211	-.777	.437	1.000
Cervical ventral horn-Precentral gyrus	-12.200	7.211	-1.692	.091	1.000
Cervical dorsal horn-Superial temporal gyrus	1.417	8.708	.163	.871	1.000
Cervical dorsal horn-Lumbar ventral horn	2.667	8.327	.320	.749	1.000
Cervical dorsal horn-Kidney	-5.467	8.327	-.657	.511	1.000
Cervical dorsal horn-Precentral gyrus	12.067	8.327	1.449	.147	1.000
Superial temporal gyrus-Lumbar ventral horn	1.250	7.649	.163	.870	1.000

Superial temporal gyrus-Kidney	-4.050	7.649	-.530	.596	1.000
Superial temporal gyrus-Precentral gyrus	10.650	7.649	1.392	.164	1.000
Lumbar ventral horn-Kidney	-2.800	7.211	-.388	.698	1.000
Lumbar ventral horn-Precentral gyrus	-9.400	7.211	-1.304	.192	1.000
Kidney-Precentral gyrus	6.600	7.211	.915	.360	1.000

Each row tests the null hypothesis that the Sample 1 and Sample 2 distributions are the same.
Asymptotic significances (2-sided tests) are displayed. The significance level is .050.
a. Significance values have been adjusted by the Bonferroni correction for multiple tests.
